# Revisiting tissue tensegrity: Biomaterial-based approaches to measure forces across length scales

**DOI:** 10.1063/5.0046093

**Published:** 2021-10-01

**Authors:** Christina-Marie Boghdady, Nikita Kalashnikov, Stephanie Mok, Luke McCaffrey, Christopher Moraes

**Affiliations:** 1 Department of Chemical Engineering, McGill University, Montréal, Québec H3A 0C5, Canada; 2 Rosalind and Morris Goodman Cancer Institute, McGill University, Montréal, Québec H3A 1A3, Canada; 3 Division of Experimental Medicine, McGill University, Montréal, Québec H4A 3J1, Canada; 4 Gerald Bronfman Department of Oncology, McGill University, Montréal, Québec H4A 3T2, Canada; 5 Department of Biomedical Engineering, McGill University, Montréal, Québec H3A 2B4, Canada

## Abstract

Cell-generated forces play a foundational role in tissue dynamics and homeostasis and are critically important in several biological processes, including cell migration, wound healing, morphogenesis, and cancer metastasis. Quantifying such forces *in vivo* is technically challenging and requires novel strategies that capture mechanical information across molecular, cellular, and tissue length scales, while allowing these studies to be performed in physiologically realistic biological models. Advanced biomaterials can be designed to non-destructively measure these stresses *in vitro*, and here, we review mechanical characterizations and force-sensing biomaterial-based technologies to provide insight into the mechanical nature of tissue processes. We specifically and uniquely focus on the use of these techniques to identify characteristics of cell and tissue “tensegrity:” the hierarchical and modular interplay between tension and compression that provide biological tissues with remarkable mechanical properties and behaviors. Based on these observed patterns, we highlight and discuss the emerging role of tensegrity at multiple length scales in tissue dynamics from homeostasis, to morphogenesis, to pathological dysfunction.

## I. INTRODUCTION

The human body is a dynamic and self-stabilizing structure formed through intricate connections between hierarchical building blocks. The mechanical structure of intra- and extracellular proteins, cells, and tissues plays a key role in achieving structural stability in response to widely varying mechanical challenges, while simultaneously enabling biological systems to actively transduce and respond to these mechanical stimuli.^1–4^ These remarkable capabilities arise from architectures that span nanometer to centimeter length scales and intimately link proteins, cells, tissues, and organs.

Cell-generated forces play critical roles in virtually all biological processes, including cell migration,^5^ tissue morphogenesis,^6–10^ muscle contraction,^11,12^ wound healing,^13^ and cancer invasion^14,15^ among others. Dysregulation of these forces often correlates with disease onset and progression,^16^ and these findings have prompted the development of novel force quantification techniques to better understand the mechanics of morphogenesis and pathogenesis. Traditional techniques, such as laser ablation, force inference, or micromachined force transducers, present several critical limitations.^17^ First, they do not allow real-time force measurements within the same tissue due to the destructive nature of the technique. Second, they offer limited sensitivity of read-outs and cannot resolve the magnitude of different types of forces. Finally, they can rarely be employed within *in vivo* or *in vivo*-like contexts, resulting in unclear translational relevance.

Biomaterials can be defined as nonviable materials, natural or manufactured, designed to interact with biological systems as part of a living structure or medical device.^18,19^ Among many other functionalities, biomaterials can be carefully designed to measure forces generated by and within cells and tissues. Recently, biomaterial-based approaches have emerged to monitor cell-generated forces in real-time and *in vivo* at varying length scales. These technologies can measure compressive, tensile, shear, anisotropic, and isotropic stresses within highly realistic and biologically relevant contexts. Moreover, in principle, active biomaterials can be designed to apply local mechanical stimuli and actively probe biological structures while passively quantifying cell-generated forces.

**FIG. 1.**
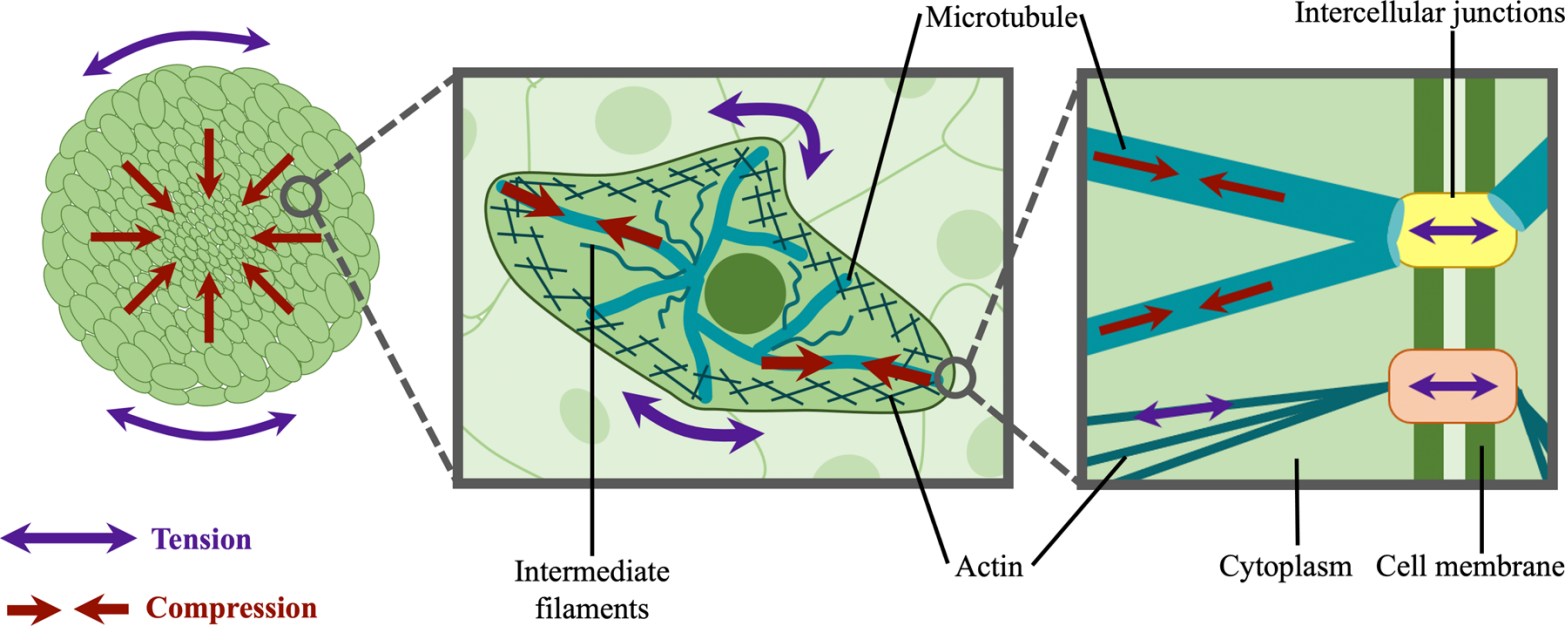
Modularity and hierarchy of tensegrity in biological tissues. A balance between compressive and tensile stresses exists from the tissue to molecular length scales. (Left panel) In microtissues such as multicellular spheroids, a tensile skin forms at the spherical boundary of microtissues while compressive stresses build up towards the core, maintaining a stable spherical structure. Inwardly directed arrows indicate compressive forces acting on core cells as a result of peripheral tension. (Center panel) At the cellular level, the active contractile forces generated by actin filaments create tension in the cytoskeletal network and cell membrane, which is resisted by microtubules and nuclei bearing a counterbalancing compressive load; thereby providing a defined cell shape and architecture. (Right panel) Molecularly, microtubule subunits are subjected to compressive stresses, while intercellular junctions and focal adhesion complexes connected to the actin network are typically under tensile load in contractile cells. These junctions transmit forces to neighboring cells, demonstrating the modular and hierarchical nature of tissue tensegrity.

Local tissue architecture and integrity is maintained by a delicate balance of forces generated through various mechanisms and by various organizing units in the body. Tensional integrity, frequently referred to by the portmanteau tensegrity, uses both compressive and tensile stresses to create a stabilizing prestress, which allows for considerable deformation under externally applied loads and restoration of the original shape after load release. This framework has been used to describe biological architectures across molecules, cells, and tissues in a hierarchical and modular fashion.^20,21^ Modular tensegrity structures assemble to form larger systems that themselves exhibit tensegrity characteristics. In this way, individual modules, such as cells, can participate in a hierarchical suprastructure, such as tissues, that are both resilient, flexible, and dynamic (Fig. 1). The hierarchical nature of these structures allow complex deformations such as shear to occur, based on the combined changes in stress distribution in networks of tensile and compressional elements, despite there being no shear-resisting formulating element in a tensegral framework.^22^ Biological systems exhibit such tensegral features across length scales, from folded proteins, to cells, to tissues, to entire organ systems. These biological structures also exist in an inherently dynamic state, responding to local stimuli and transducing biochemical and mechanical changes. Since they maintain their mechanical integrity through these transitions, it seems reasonable that tensegrity must play an important role in dynamic biological processes.

Tensegrity was first defined and discussed by Fuller in the late 1950s as an architectural principle relying on balancing continuous tension and discontinuous compression forces to provide a stably prestressed structure.^23^ While traditional tensegrity structures predominantly consider compression-bearing struts and tense cables, broader definitions have since emerged to encompass a wider selection of prestressed structures. An energy-based interpretation of tensegrity proposes stability as an equilibrium configuration which minimizes the stored elastic energy in the structure, and considers any internal and external compression components providing structural stability.^24,25^ Given the impact of tension and compression in both cellular and non-cellular components of biological systems, in this review, we consider Fuller's structural definition, but define the system more broadly so as to incorporate additional external load-bearing components as in the energy-based interpretation. This approach allows us to consider tensegrity-oriented features in the tissue microenvironment, such as extracellular matrix (ECM) architecture, hydrostatic, osmotic, or pneumatic pressure, interfacial tensions, and other components, which confer stability to the biological system. Since the microenvironment has been shown to play a critical role in tissue development and function, while pathological conditions are frequently related to microenvironmental dysregulation,^26–29^ we believe that exploring this larger reference frame through the lens of tensegrity may yield interesting insights into biological processes of development and disease.

At the cellular length scale, the tensegral nature of biological systems has been well-defined, most notably by Ingber and colleagues, whereas tensegrity in molecular and tissue scale systems remains more speculative. Many mechanical elements contribute to the structural stability of cells, including osmotic pressure, actin contractility, filament polymerization, and cell distention through adhesions to the ECM or to neighboring cells. Among other components, microtubules balance cytoskeletal prestress with a decreasing contribution during cell spreading.^30^ While microtubules have been found to bear some compressive loads, this is minor compared to extracellular traction forces,^31^ and other structures such as the nucleus may play a larger role in resisting tension.^32–35^ Interestingly, the nucleus itself also exhibits tensegral features, as it is stabilized by its internal components, nuclear pressure, and the nuclear envelope, further demonstrating the modular and hierarchical arrangement of tensegrity components in a cell.^36^ Cellular tensegrity, hence, acts as a stabilizing feature that permits actuation and deformation, giving rise to cellular architecture, stability, and dynamics.^36–38^ Higher levels of biological organization also show tensegral patterns of stabilization in tissues, organs, and the whole body,^39,40^ and experimental observations have been interpreted within the framework of tensegrity to illustrate mechanotransductive and morphogenetic processes at the cellular level.^41^ However, quantifying such mechanical forces contributing to tissue structure and dynamics has proven to be a considerable technical challenge due to the spatial scale, time-dependency, and range of forces present in biological systems.

In this review, we summarize recent biomaterials-based methods to study cell-generated forces at the molecular, cellular, and tissue scale within biological tissues. We also revisit the implications of the tensegrity model at the molecular, cellular, and tissue length scales, based on the read-outs of cell-generated forces. Finally, we highlight how a tensegral balance between forces plays an important role in stabilizing local architecture and generating morphological and biological responses to microenvironmental stimuli, and theorize that large imbalances in cell-generated forces may lead to disease.

## II. MEASURING FORCES AT THE MOLECULAR LENGTH SCALE

**FIG. 2.**
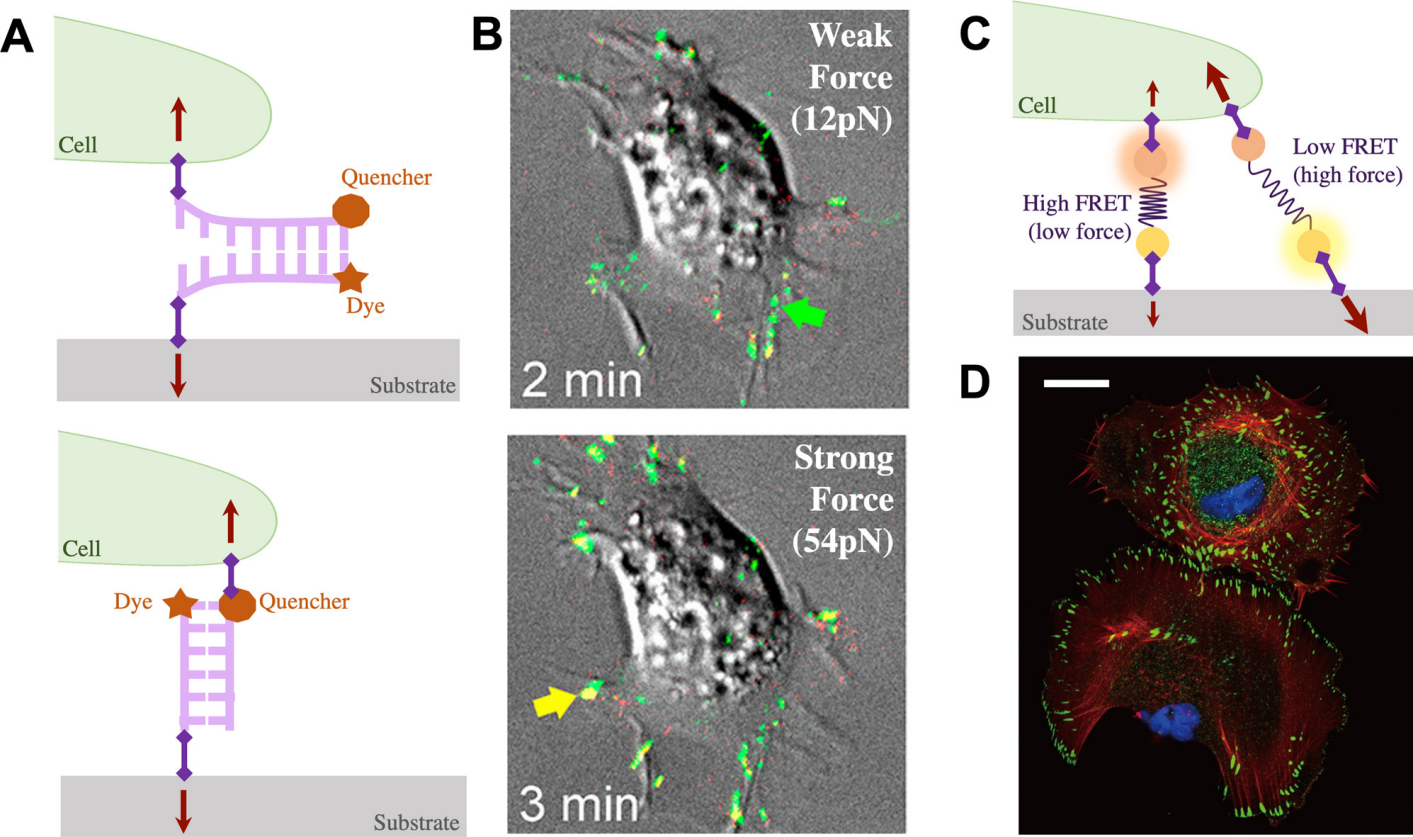
Molecular techniques for measuring cell-generated forces. (a) Schematic of fluorescent dye-quencher pair probe labeling strategies for a tension gauge tether assay to visualize and measure molecular tension in quasi-real time (adapted from Ref. 42) Purple diamonds indicate attachment to the substrate through biotin–neutravidin interactions, while an RGD peptide linker allows cell attachment through integrins. Linker configuration can be designed to measure weak forces (top) or strong forces (bottom). (b) Tension gauge tethers have a defined physical rupture force of the double-stranded DNA, as published in Jo *et al.*^42^ Reprinted with permission from Jo *et al.*, ACS Biomater. Sci. Eng. **5**(8), 3856–3863 (2019). Copyright 2019 American Chemical Society. (c) Schematic of sensor molecules made of a FRET donor (yellow circle) and acceptor (orange circle) pairs that are separated by an elastic peptide spring (adapted from Ref. 43) Sensors are anchored to a biotinylated polyethylene glycol brush on a coverslip and an RGD domain is added for integrin binding. (d) Upon integrin binding to the RGD sequence, cells can apply a load generated by the cytoskeleton to pull the sensor and the force can be measured based on FRET decay, as published in Morimatsu *et al.*^43^ (blue: nucleus; red: actin; green: paxillin). Scale bar: 25 *μ*m. Reprinted with permission from Morimatsu *et al.*, Nano Lett. **13**(9), 3985–3989 (2013). Copyright 2013 American Chemical Society.

Measuring forces at the molecular scale requires similarly sized probes. In this section, we consider tools that have recently been developed by engineering DNA (deoxyribonucleic acid) and proteins to provide unique force-readout capabilities (Fig. 2; Table I). Here, we highlight how these strategies provide unique insight into molecular length-scale mechanobiology and tensegrity. We also draw specific attention to the limitations of such approaches, particularly in terms of their ability to quantify compressive forces, as well as their efficacy and utility in advanced tissue engineered models.

**TABLE I.** Biomaterials for measuring cell-generated forces at the molecular scale.

Technique	Biomaterial	Readout method	Readout range	Force type	Pros	Cons	Ref.
DNA probes	DNA oligo-nucleotides (double-stranded or single-stranded hairpin structures)	Fluorescence when fluorophore-quencher pairs are pulled apart by specific forces	10–100 pN	Tension, shear	• Customizable design to measure specific forces	• Discrete measurements only	44,45
					• High resolution and specificity	• Little information on force orientation	
Protein probes	FRET protein fusions	Fluorescence when FRET pairs are pulled apart by specific forces	1–100 pN	Tension	• Same sensor can measure a continuous range of forces	• Difficult calibration	46–48
					• High resolution and specificity	• Limited continuous measurements over time	

### A. DNA probes

DNA-based techniques use DNA oligonucleotide sequences that can be custom-synthesized to measure molecular forces between cells and their substrates within the 10–100 pN range.^49^ Briefly, complementary regions of DNA can be designed to resist a defined amount of force, before unzipping and detaching from each other in a double-stranded format^45,50^ or extending secondary structures such as hairpin loops in a single DNA strand [Fig. 2(a)].^42,44^ The rupture force is dependent on the length and nucleotide composition of the DNA sequence as well as the position along the length of DNA where the rupture force is being applied. This rupture point can be designed to include protein tags like biotin or simple polypeptide chains like arginine–glycine–aspartate (RGD), which serve as attachment points to cells or functionalized surfaces. In this configuration, DNA probes can act as threshold tension limiters to manipulate the strength of cell adhesions to surfaces. If the rupture threshold is exceeded and the tether breaks, the cell is unable to spread, and tension-dependent downstream signaling is disrupted. Hence, this system limits the forces that cells can generate, which can, in turn, be used to manipulate cell adhesion, morphology, and transcription factor nuclear localization.^45,50^ Interestingly, fluorophore and quencher pairs can be incorporated into these DNA structures such that unzipping results in a fluorescent signal that can be tracked to monitor when traction forces exceed defined thresholds in real-time [Fig. 2(b)].^42,44,51,52^

These cell-substrate DNA-based sensors have been used to control and quantify the tension between an integrin–ligand bond,^42,52^ tension requirements to create a focal adhesion,^53^ and the tension required to activate notch signaling.^45^ The interplay between these forces regulates cell spreading, focal adhesion formation, morphology, and ultimately function.^44,47,50^ DNA probes, thus, provide a unique way to quantify tensional stresses contributing to molecular tensegrity structures in protein–protein interactions and binding, ultimately regulating cell spreading, morphology, and function. However, the stringent imaging and surface-modification requirements to make these single-molecule measurements make it quite challenging to extend these studies into three-dimensional (3D) culture materials, and these techniques are often limited to isolated cells on a 2D surface.

### B. Protein probes

Molecular forces can be directly read out by designing protein-based probes that use fluorescence resonance energy transfer (FRET) technology to optically image forces [Figs. 2(c) and 2(d)].^43^ FRET uses pairs of donor and acceptor fluorescent proteins to readout the proximity between fluorescent pairs. Excitation of the fluorescent donor protein transfers energy to a nearby acceptor protein with a similar resonance frequency, causing it to fluoresce if the pair of proteins are sufficiently close. To measure forces, intracellular fusion proteins can be designed to include donor and acceptor protein pairs separated by an elastic polypeptide linker within the protein of interest.^46^ This linker domain acts like a spring tethering the donor and acceptor fluorescent proteins together. Therefore, when there is tension across the protein of interest, the donor and acceptor molecules are separated and shift their emission spectra, which correlates with the force required to stretch the linker region.

Protein-based biomaterials for mechanical measurements have been validated and compared against single-molecular force spectroscopy techniques such as optical tweezers, magnetic tweezers, and atomic force microscopy. While the details of these techniques are beyond the scope of this review, single molecule techniques have been applied to biological molecules to quantify protrusive cell forces,^54^ adhesion forces between cells^55^ and between ligand–receptor pairs,^48,56,57^ force required to unfold proteins,^58^ DNA strands,^59,60^ and polysaccharides.^61^ They also provided early details about forces exerted on and by actin filaments^62^ as well as morphological effects on cells under applied loads.^63^ Thus, a variety of molecular scale forces play an integral role in regulating cell function and applying the tensegrity model may facilitate interpreting the result of their interactions. Unfortunately, these techniques do not provide any information on compressive stresses within these systems, which has been shown to play important roles in morphogenesis, cancer progression, and stem cell differentiation.^64^ With further refinement, FRET-based sensors provide a unique opportunity to study compressive stresses since their read-outs can capture the nearing of donor and acceptor proteins, as opposed to DNA probes. Therefore, further advances in identifying tensegrity structures requires novel methods for quantifying compressive stresses involved in homeostasis and disease progression.

### C. Molecular tensegrity

Molecular scale tensegrity structures have been significantly less studied in comparison to cellular structures, but the concept has provided a model for rationalizing protein folding^65^ and networks of hydrogen bonding in globular proteins.^66^ Rigid domains such as alpha helices and beta sheets act as compressive load bearing structures within the protein, while flexible regions are under tensile stress.^39,67^ Additionally, structural prestress can be inferred by shape instabilities occurring after cleavage.^68^ DNA structures have also been engineered to assemble into prestressed tensegrity structures with double helices under compression and single helices under tension.^69,70^ However, most of this evidence has arisen from *in silico* modeling, and supporting experimental data remains sparse. Thus, biomaterial-based approaches to quantifying cell-generated forces at the molecular scale provide a unique opportunity to uncover molecular tensegral patterns.

Designing molecules present at the anchoring sites between cells and their surroundings can, hence, provide considerable information on tensile forces around a cell as well as a robust means of controlling cell shape and overall tissue architecture. Hierarchically organized and modular tensegrity structures are present throughout this process. Since a cell's ability to probe its mechanical microenvironment largely depends on receptor binding, these conformational changes to molecular tensegrity redistribute forces upon loading and therefore tightly couple mechanical and chemical signaling mechanisms at this length scale. Proteins, such as ATP (adenosine triphosphate) synthase and dynein, have been modelled as tensegrity structures to simulate conformational shifts involved in phosphorylation and sperm motility, respectively.^67^ A tensile network of hydrogen bonds was shown to provide a stabilizing prestress, while stiff structures like beta sheets and alpha helices bear compressive loads and other subunits lengthen under tension. The tensegral balance in forces and prestress within the molecules were found to be crucial for proper function.^67^ Therefore, it can be hypothesized that, such as ATP synthase and dynein, other molecular components of cells can behave as a tensegrity structures, where mechanically activated conformational changes are facilitated by tensile and compressive bearing elements as well as molecular prestress. Hence, molecular tensegrity plays a crucial role in force-activated conformational shifts, providing a basis for mechanobiological force-feedback loops to stabilize and control cellular, and ultimately tissue architecture.

## III. MEASURING FORCES AT THE CELLULAR LENGTH SCALE

**FIG. 3.**
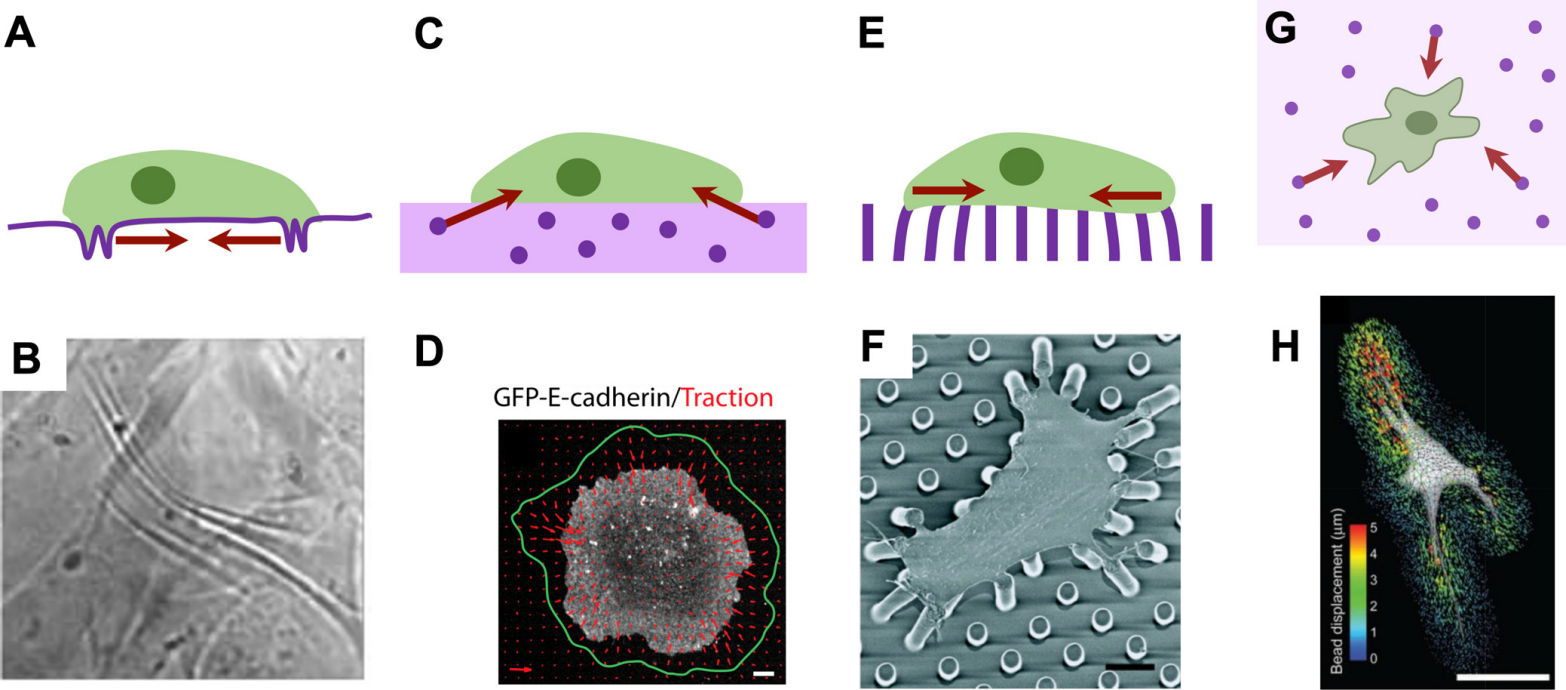
Techniques for measuring biological forces at the cellular length-scale. Arrows indicate biomaterial deformations induced by cell-generated forces. (a) Adherent cells on thin silicone rubber films contract and wrinkle the substrate. (b) Fibroblasts plated on a thin film visibly wrinkle the substrate. Field of view is 190 × 176 *μ*m^2^. Reprinted with permission from Yu *et al.*,^72^ Biomed. Opt. Express **3**(1), 153 (2011). Copyright 2011 Author(s), licensed under the Creative Commons Attribution-Noncommercial-No Derivative Works 3.0 Unported License. (c) Traction force microscopy quantifies contractile cell forces in 2D by tracking the displacement of embedded fiduciary markers in compliant and mechanically characterized substrates. Out-of-plane traction forces can be quantified by tracking upward displacements with advanced microscopy techniques. (d) Madin-Darby Canine Kidney (MDCK) cells expressing GFP-E cadherin exert tractions on a collagen-coated polyacrylamide substrate. Red arrows indicate traction vectors and the green line indicates the considered area for computing total traction force. Scale bar = 5 *μ*m. Reprinted with permission from Maruthamuthu *et al.*,^76^ Proc. Natl. Acad. Sci. **108**(22), 4708 (2011), Copyright 2011 National Academy of Sciences, USA. (e) Micropillar arrays quantify contractile cell forces in 2D by tracking pillar deflection. (f) A smooth muscle cell exerts traction forces on top of fibronectin-coated pillars. Scale bar = 10 *μ*m. Reprinted with permission from Tan *et al.*,^77^ Proc. Natl. Acad. Sci. 100(4), 1484 (2003). Copyright 2003 National Academy of Sciences, U.S.A. (g) 3D traction force microscopy quantifies cell forces in 3D by tracking the displacement of embedded fiduciary markers in compliant and mechanically characterized matrices. (h) A fibroblast in a 3D hydrogel exerts traction forces in 3D, which are tracked by mapping bead displacements. Scale bar = 50 *μ*m. Reprinted with permission from Legant *et al.*,^78^ Nat. Methods **7**, 969 (2010). Copyright 2010 Springer Nature.

Adherent cells will actively bind to surrounding materials and produce cell traction forces strong enough to deform a sufficiently flexible material. Cells grown on silicone rubber films, for example, will wrinkle the substrate, which allowed Harris *et al.* to directly visualize cellular contractile behavior [Figs. 3(a) and 3(b)].^71–73^ Contractility-associated integrin clustering and actin fiber bundling^74^ suggest that traction forces depend on both the tensegrity-like cytoskeleton and on external stimuli. Traction forces are primarily generated through actin–myosin interactions, and in this model, the cytoskeleton is stabilized by myosin-generated tensile stresses distributed across various load-bearing cytoskeletal protein structures (e.g., actin and microtubules).^25^ Measuring forces at the cellular scale requires tracking the cell-generated force-induced displacements of engineered landmarks on or within mechanically characterized substrates fabricated from diverse native or synthetic materials (Table II, Fig. 3). While the techniques to quantify cell-generated forces have been thoroughly reviewed elsewhere;^17,49,75^ here, we specifically focus on their contributions in understanding the role of tensegrity in cellular biological architectures.

**TABLE II.** Biomaterials for measuring traction forces at the cellular scale.

Technique	Scale	Biomaterials	Readout method	Readout range	Pros	Cons	Ref.
2D films	Cellular	Silicone rubber	Wrinkling of film	1–10^4^ nN	• Direct visualization of contractile cell behavior	• Difficult calibration, fabrication, and quantification	71,73
					• Quantitative measurements possible		
2D TFM substrates	Cellular or epithelial sheets	Engineered elastomers (silicone, PDMS, gelatin, polyacrylamide) or natural matrices (fibrin, collagen)	Embedded fiduciary marker displacement in horizontal plane	1–10^4^ Pa	• Quantitative and directional assessment of forces	• Computationally intensive unless a reference-free adaptation is used	75,78–86,91,97,98
					• Tunable linear mechanical properties of engineered substrates	• Limited to 2D tractions	
						• Non-linear mechanical properties require complex constitutive models	
3D-like (2.5D) TFM substrates	Cellular	Polyacrylamide, PEG	Embedded fiduciary marker displacement in horizontal and lateral planes	1–10^4^ Pa	• Quantifies out-of-plane tractions relevant in 3D microenvironments	• More computationally intensive than 2D TFM	93
						• Still 2D microenvironment	
2D micropost arrays	Cellular or epithelial sheets	PDMS	Pillar deflection	10^−2^–10^2^ nN	• Less computationally intense	• Topography does not resemble physiological conditions	77,114–116
					• Customizable substrate mechanical properties by adjusting post geometry	• Limited deflection of posts possible	
						• Discrete measurements only	
3D TFM substrates	Cellular	Engineered matrices (Matrigel, PEG, agarose, hyaluronic acid hydrogel, dextran, etc.), natural matrices (collagen, fibrin, etc.), and composite blends	Embedded fiduciary markers in all planes	10–10^4^ Pa	• More physiologically relevant	• Computationally intense	78,132–136
					• Tunable linear mechanical properties of engineered matrices	• Non-linear mechanical properties of natural matrices complexifies quantification	
						• Microenvironment mechanical properties change during contraction due to remodeling	

### A. On 2D flat substrates

In traction force microscopy (TFM), fiducial markers are introduced into mechanically defined compliant substrates and tracked to quantify both the magnitude and direction of cell traction forces [Figs. 3(c) and 3(d)].^76,79–87^ Initially performed with silicone rubber substrates,^88,89^ this technique expanded to include increasingly more biologically relevant materials such as polyacrylamide,^90,91^ gelatin,^92^ agarose,^93^ fibrin,^94^ and collagen^95,96^ hydrogels. Polyacrylamide gels have been widely used in TFM as they are linearly elastic across a wide range of strains, which facilitates traction force computations, and their stiffness can be easily tuned by altering polymer composition. Patterning fiducial markers further simplifies traction force measurements and alleviates computational load.^97,98^ Additionally, substrates can be stretched to induce predetermined strains on adhered cells, while measuring their mechanical response as traction forces and internal displacements of endocytosed beads.^99^ In this way, cellular prestress can be assessed at the subcellular length scale.

The mechanical behavior of cells on surfaces demonstrates characteristics of tensegrity in which tensile forces are balanced against compressive loads. Cells attached to gel surfaces establish mechanical homeostasis by using their endogenously generated cytoskeletal tension, or prestress, to pull in when generating contractile forces, effectively inducing a compressional component in the underlying substrate. Traction forces correlate with cytoskeletal tension,^25,99,100^ and disruption of individual cytoskeletal tension-generating stress fibers offsets the balance between internal tension and external traction, causing a net reduction in traction forces.^101^ Actin stress fibers act as tension-bearing components in the cytoskeletal tensegrity structure, and locally compromising these fibers would hypothetically result in the redistribution of forces within the structure. Therefore, observing a decrease in contractile forces upon disruption of load-bearing elements supports the applicability of the tensegrity model at the cellular scale. Moreover, cytoskeletal stiffness is observed to proportionally increase with actin contractility,^25,41^ and internal shear modulus increases with cellular prestress.^99^ Both observations are consistent with tensegral behavior, where cohesively increasing structural prestress and stiffness is an intrinsic property of tensegrity structures.

Cells also exhibit larger traction forces on stiffer substrates, further suggesting that external mechanics exogenously promote the development of cell prestress as well as the formation of stress fibers and focal adhesions.^102–104^ Alterations in the mechanical environment have been shown to play a role in pathogenesis, suggesting that there is a delicate balance in cellular prestress that must be maintained for healthy function. For example, on substrates of similar stiffness to a healthy myocardium, cardiomyocytes show an aligned sarcomere structure and generate greater mechanical force, while on stiffer substrates reminiscent of disease situations, they lose their sarcomeres, form stress fibers and produce weaker contraction forces.^105^ These mechanically driven disease-like phenotypes have been observed in a wide variety of tissue systems ranging from the placenta^106,107^ to cancer.^103,108^

TFM can also be applied to multicellular sheets, to measure traction stresses exerted on the substrate, and also to quantify cell–cell forces.^109–111^ Multicellular TFM provides additional supporting evidence that cell sheets can behave as modular tensegrity structures, where cells act as individual components in a cohesive suprastructure. Maximal stresses appear to ripple across the whole sheet through cell–cell junctions and direct migrations.^109^ These stress waves also play an important role in tissue expansion, as peripheral cells undergo “unjamming” to be more motile at the border.^112^ Exposing sheets to additional loads in the form of applied shear flow also increases cytoskeletal and intercellular tension, regulating the assembly of cell–cell junctions.^113^ These observations together highlight the formation of hierarchical tensegrity structures from individual cells to cell sheets and suggest that tensegrity may be a defining characteristic allowing force transmission across multicellular sheets.

### B. On 2D micropillar arrays

The capabilities of TFM can be greatly expanded by fabricating silicone rubber substrates into arrays of flexible micropillars, pioneered by Tan *et al.*, which can deflect under the action of cell-generated traction forces [Figs. 3(e) and 3(f)].^77,114–116^ Micropost array systems bypass the challenges associated with tracking the displacement of beads and efficiently determine cell traction forces by measuring the deflection of individual pillars.^117^ Furthermore, the stiffness, length, and geometry of the microposts can also be manipulated to control the mechanical microenvironment presented to cells. For example, shorter posts present stiffer cues for cultured cells,^118^ while ellipsoidal microposts can recreate anistropic mechanical properties.^119^ Post diameters can also be decreased to have higher resolution mapping of traction forces and focal adhesion dynamics. On submicron pillars, forces are exerted between neighboring pillars, generating apparent local contraction events that produce small nano-scale displacements, akin to cells pinching posts to sense the substrate's rigidity.^120^ Similar to monolayer TFM, stresses within a cell sheet, as quantified on micropost arrays, are higher at tissue edges^121^ and are transduced through cell–cell junctions^122^ in a modular fashion.

Micropost arrays can also be actuated to subject cells to external mechanical stimulation through local magnetic micropost deflections^115,123–126^ or through global mechanical^127,128^ and vacuum-driven^129,130^ stretching of the whole array. External application of tension by stretching provides a unique opportunity to probe at cellular tensegrity structures and quantify mechanical effects in the form of traction forces. Some cells remain unaffected by local actuation while some generate reduced tractions at the cell periphery.^115^ Smooth muscle cells, on the other hand, appear to contract more upon stimulation, leading to an apparent global force reinforcement.^124^ They also appear to exhibit a biphasic response where, during global stretching, they initially contract more at the cell poles to resist rapid cell deformation, but then release tension in their cytoskeleton and soften by reorganizing themselves and allowing relaxation.^129^ In particular, to return to mechanical homeostasis after stretching, fibroblasts utilize both focal adhesions and cytoskeleton tension mechanisms,^128^ which are intrinsically related to cellular tensegrity. Such time-dependent effects of external mechanical stimulation suggest that there is a dynamic nature to biotensegrity structures, which likely arises from the viscoelastic and plastic properties of the cytoskeleton.

### C. In 3D matrices

Although monolayer TFM and micropost array systems have proven invaluable in understanding the structural aspects of the dynamic prestressed cell cytoskeleton as well as its response to active perturbations, they are inherently limited to measuring contractile and shear stresses in 2D. Cells have the capability of generating out-of-plane traction forces in 3D contexts, and 3D culture is known to significantly affect cell function.^78,131–136^ However, obtaining 3D traction forces within conventional 3D culture presents significant challenges. Cell-induced 3D deformations can be tracked in collagen,^134,137^ fibrin,^137–140^ and matrigel^135^ matrices via the movement of fiduciary markers or the distortion of the fibrous matrix network. The traction forces driving these deformations have only recently been quantified due to difficulties associated with characterizing the complex mechanical properties of most native matrices. Not only do natural matrices exhibit non-linear strain-stiffening material properties but also their characteristics change dynamically near cells as they pull, stiffening and irreversibly remodeling the matrix.^133,137,138,141^ Recently, constitutive laws have been developed, allowing the reconstruction of cell traction stress fields from matrix fiber displacements as obtained using confocal reflection microscopy^136,142^ or from bead displacements acquired with optical coherence microscopy [Figs. 3(g) and 3(h)].^143^

To facilitate the measurement of 3D traction forces, engineered matrices such as polyethylene glycol diacrylate,^78^ dextran methacrylate^144^ or polystyrene^145^ with well-defined mechanical properties can be used. Although these scaffolds do not replicate *in vivo*-like conditions to the same extent as natural matrices, cells grown in these synthetic gels still behave realistically. They produce larger inward tractions in stiffer matrices that increase at the cell periphery and are localized to the cell's slender protrusions.^78^ In addition, the mechanical and architectural characteristics of these synthetic matrices can be easily tuned to more precisely study the influence of the local microenvironment on cell behavior. Adherent cells seeded within stiff dextran methacrylate gels with aligned fibers migrate continuously following a 2D migration mechanism. However, when cells are grown in disordered deformable matrices, they migrate using a slingshot mechanism where they contract the matrix and store elastic energy within it to rapidly jump forward, following matrix recoil due to loss of adhesion at the cell rear.^144^ The extracellular scaffold can be interpreted as a modular tensegrity structure to the cell, where matrix fibers bear tensile loads which counterbalance hydrostatic or cellular compressive stresses, preventing the structure from collapsing on itself. Cells are attached to their external matrix through focal adhesions, which are also linked to their tensegral cytoskeleton, effectively linking them to form a tensegral suprastructure. Variations in neighboring components induce tensegrity structures to redistribute structural loads to regain stability, resulting in the propagation of a local response. Therefore, the observed change in cellular behavior in response to alterations in the surrounding matrix suggest that the ECM and cells act as modular tensegrity structures to maintain the construct's stability.

Interestingly, and similar to molecular force sensors, these cellular scale methods also generally quantify tensile forces, and measuring piconewton-scale compressive stresses remains a challenge. To provide compressive stress readouts, compliant sensors can be designed to sustain compressive loads and deform in a measurable ways. Recently, subcellular hydrogel microparticles have been used to quantify compressive stresses during phagocytosis,^146^ showing promise for future applications in morphogenesis, cancer progression, and stem cell differentiation.^64^

## IV. MEASURING FORCES AT THE TISSUE LENGTH SCALE

**FIG. 4.**
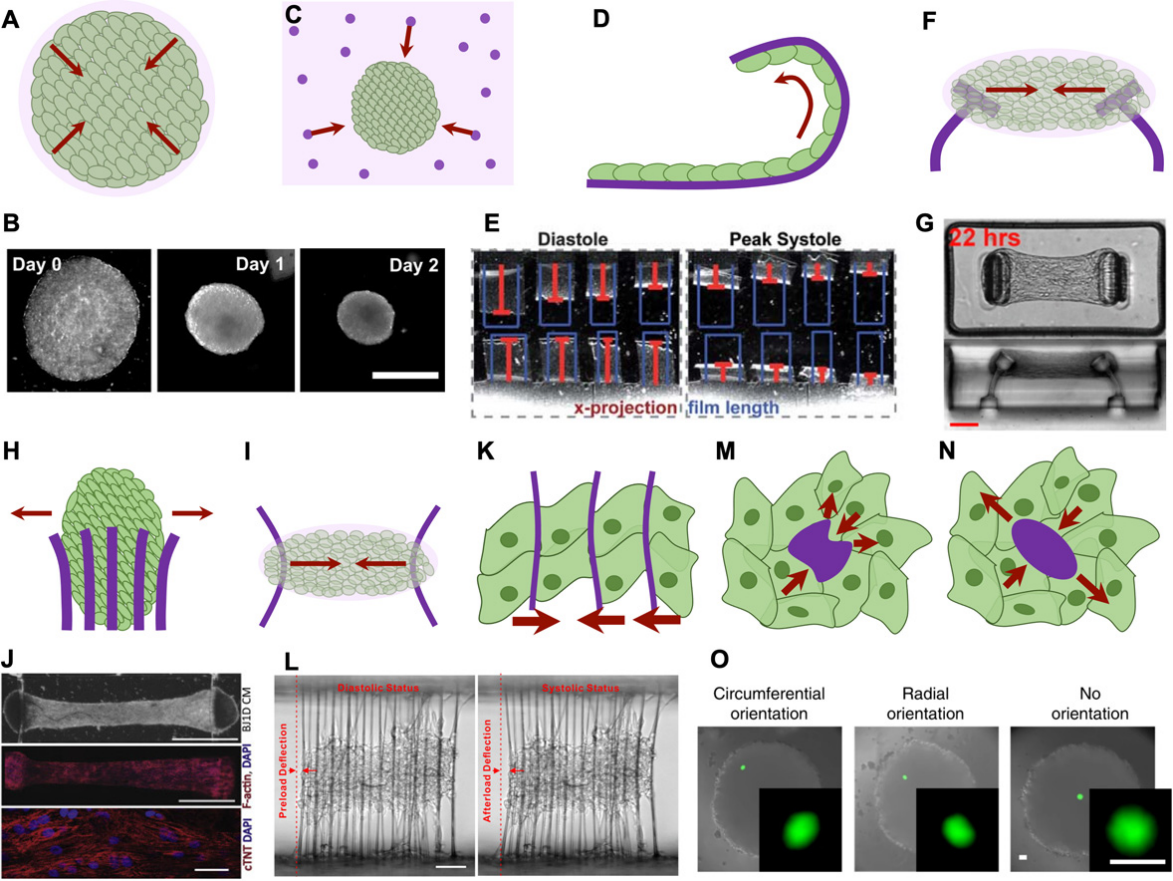
Techniques for measuring tissue-scale forces. Arrows indicate biomaterial deformations due to cell-generated forces. (a) Cell-laden matrices contract due to cell-generated forces. (b) Microdroplets of collagen are contracted by MC 3T3 cells. Scale bar = 1 mm. Reprinted with permission from Moraes *et al.*,^147^ Biomaterials, **34**(37), 9623–9631 (2013). Copyright 2013 Elsevier. (c) Patterned microtissues are embedded within a mechanically characterized matrix with fiduciary markers and forces are quantified by tracking their displacements. (d) Thin film cantilevers curl up under contractile forces exerted by adherent cell sheets which are quantified. (e) Cardiomyocyte sheets are seeded onto thin PDMS films, which curl under diastolic and systolic contraction. Contractile stresses are computed from the relative x-projection of the curled film to the initial film length. Republished with permission from Grosberg *et al.*, Lab Chip, 11(24), 4165 (2011);^148^ Copyright 2011 Clearance Center, Inc. (f) Embedded micro-cantilevers induce prestress within the self-assembled tissue constructs and the deflection of cantilevers are correlated with contractility. (g) Self-assembled microtissues of NIH 3T3 cells in collagen contract and bend embedded PDMS cantilevers. Scale bar = 100 *μ*m. Reprinted with permission from Legant *et al.*,^149^ Proc. Natl. Acad. Sci. 106(25), 10097 (2009). Copyright 2009 National Academy of Sciences, USA. (h) Pillars deflect as the spheroid grows and quantifies outwards growth forces. (i) Parallel wires hold the self-assembled tissue construct in place and deflect inwards due to contractile forces. (j) Cardiac microtissues derived from BJ1D stem cells self-assemble around POMaC wires, which deflect under generated contractile loads. F-actin and troponin-T staining show cardiomyocyte and sarcomeric contractile protein alignment, which are necessary for proper tissue function. Top and middle scale bar = 1 mm. Bottom scale bar = 30 *μ*m. Reprinted with permission from Zhao *et al.*,^150^ Cell **176**(4), 913–927 (2019). Copyright 2019 Elsevier. (k) Filamentous matrices deflect like parallel wires, but are smaller and quantify contractile forces at the single-cell scale in tissues. (l) Cardiac microtissues passively contract 5 *μ*m OrmoClear™ fibers in diastolic status, and are actively contractile in systolic status. Scale bar = 100 *μ*m. Reprinted with permission from Ma *et al.*,^151^ Nat. Biomed. Eng. **2**, 955 (2018). Copyright 2018 Springer Nature. [(m) and (n)] The deformation of (m) oil or (n) hydrogel microdroplets injected into tissues allows measurement of cell-generated anisotropic forces (m) or isotropic forces (n). (o) Circumferentially oriented hydrogel micropdroplets indicate that there are tensile stresses at the periphery of fibroblast spheroids, while compression radially compresses droplets. Scale bar = 50 *μ*m. Reprinted with permission from Lee *et al.*, Nat. Commun. **10**, 144 (2019).^152^ Copyright 2019 Author(s), licensed under a Creative Commons Attribution (CC BY) license.

While cellular length scale tensegrities have been quite well-established, understanding tensegrity at intermediate length scales between cells and organs remains challenging, primarily due to technical limitations in quantifying forces within small tissues. Forces at the tissue scale are more complex than the simple sum of cellular scale forces, as additional factors such as collective behaviors, spatial profiles, and supracellular elements must be considered. Measuring forces at the tissue length scale generally requires some knowledge of the mechanical properties of the tissue, which typically depends on tissue state and composition, and are usually non-linear and time-variant. Furthermore, these properties can be highly heterogenous, particularly at the length scale of individual cells. Hence, recent strategies to resolve tissue-scale forces have involved designing tissues with small regions of precisely defined properties, and measuring the deformation of these defined zones to quantify generated forces. These techniques can be broadly split into two categories: tissue-scale measurement of average forces [Table III, Figs. 4(a)–4(j)] or cellular-scale measurement of cell forces within tissues [Table IV, Figs. 4(k)–(o)].

**TABLE III.** Biomaterials for measuring cell-generated forces at the tissue scale.

Technique	Biomaterials	Readout method	Readout range	Force type	Pros	Cons	Ref.
Contracting cell-laden gels	Natural ECM (collagen)	Observed contraction of microtissue	1–10 mN	Contraction	• Resolves tissue-relevant contractile forces in 3D	• Non-linear mechanical properties of ECM gels make direct force quantification difficult	147,153–155
					• Quantifiable contraction in physiologically relevant microenvironment	• No spatial resolution within the tissue	
					• Self-assembled tissue construct		
3D tissue TFM substrates	Natural ECM (collagen) and engineered matrices (silk-collagen hybrid)	Embedded fiduciary markers in all planes	1–10^3^ *μ*N 10–10^3^ Pa	Traction	• Physiologically significant microenvironment and geometry	• Computationally intense	158,163,164
					• Resolves spatial profiles related to tissue geometry and function	• Quantifies overall tissue traction forces, no information on internal spatial profiles	
						• Non-linear mechanical properties of native matrices complexifies quantification	
Thin film cantilevers	PDMS, alginate, gelatin, dextran, collagen	Thin cantilever buckling and deflection	0.1–100 kPa	Contraction	• Direct quantification of tissue contractility	• Challenging fabrication and imaging	148,168,169
					• High-throughput measurements possible	• Only for cell sheets or thin tissue not embedded in ECM	
						• Little spatial information of spatial profiles	
Embedded micro-cantilevers	PDMS	Cantilever buckling and deflection	0.1–10 *μ*N 0.1–10 kPa	Contraction	• Self-assembled tissue construct	• Only quantifies uniaxial contractile stresses	149,183,185, 197,206
					• Direct contractility quantification of 3D microtissues without need for high computation power	• Limited spatial profiling of internal tissue forces	
					• High-throughput measurements possible	• Limited deflection range of posts and possibility for tissue to fall off cantilevers	
3D micropost arrays	PDMS	Post deflection	100–300 nN	Growth-induced compressive stresses	• Direct growth force quantification of 3D microtissues	• Confined tissue as growth continues	189
						• Limited spatial profiling of internal tissue stresses	
Wires	POMaC/PDMS, PDMS/titanium composites	Wire deflection	1–100 *μ*N	Contraction	• Robust hold on microtissue preventing falling off from device	• Only quantifies uniaxial contractile stresses	150
					• Direct contractility quantification of 3D microtissues	• No spatial profiling of internal tissue forces	

**TABLE IV.** Biomaterials for measuring cell-scale forces within tissues.

Technique	Scale	Biomaterials	Readout method	Readout range	Force type	Pros	Cons	Ref.
Filamentous matrices	Cellular and cellular within tissue	Organic/inorganic hybrid polymer (OrmoClear)	Filament deflection	10–10^5^ nN	Contraction	• Quantifies contractile stresses at the single-cell level within tissues	• Computationally intense	151,195
						• Customizable geometry and patterning of mechanical properties of the microenvironment	• Requires inhomogeneous microenvironment	
							• Challenging and low throughput fabrication	
Oil droplets	Cellular within tissue	Fluorocarbon, ferrofluid	Droplet deformation	0.01–5 kPa	Anisotropic	• Measures single cell-generated anisotropic forces within tissues	• Limited upper range of force measurements due to breaking up of droplets	207,209
						• Customizable surface treatment of droplets	• Cannot quantify isotropic forces	
						• Allows spatiotemporal profiling of forces *in vivo*		
Hydrogel droplets	Cellular within tissue	Polyacrylamide, PEG, alginate	Droplet deformation	0.1–10 kPa	Isotropic (tension, compression)	• Measured single cell-generated isotropic forces within tissues	• Challenging fabrication and imaging	152,211,212,216,217
						• Customizable surface treatment of droplets	• Requires monodisperse bead fabrication or unstrained bead dimensions for quantification	
						• Allows spatiotemporal profiling of forces *in vivo*		

### A. Macro-scale bulk tissue forces

Microengineered tissues are a powerful tool to recreate and study cellular behavior in *in vivo-*like conditions, encompassing cell–ECM and cell–cell interactions. Developing such tissues typically requires the cells to remodel, reorganize and structure the tissues, through processes such as matrix contraction [Figs. 4(a) and 4(b)].^147,153–155^ Recent advances in bioprinting have allowed fabrication of more complex structures such as tissue beams, where their deformations under cell-generated stresses are used to quantify mechanical properties and stabilizing tissue behaviors.^156^ In this section, we review strategies to measure forces across such tissues, which can often be challenging due to their evolving viscoelastic nature.

#### 1. Tissue-scale 3D traction force microscopy

Collective tissue forces can be quantified by embedding complex tissues such as breast ducts^157–160^ or cancer spheroids/tumoroids^161–165^ in ECM with well-defined mechanical properties and integrated fiduciary markers to track displacement and stress fields during tissue morphogenesis [Fig. 4(c)]. Using this technique, high mechanical stress and peripheral tensile stresses were found to arise during invasion of mammary epithelial tissues into the surrounding ECM, at sites of acute tissue curvature. The increase in peripheral tension compresses the surrounding matrix and facilitates normal duct formation and outward collective cell migration.^158,160^ In pathogenic conditions, reducing the peripheral contractility of cells in the tumor decreases their invasive potential in both breast cancer^166^ and fibrosarcomas.^165^ These contrasting observations suggest an optimal tensile balance at the microtissue periphery, which is essential for maintaining homeostatic architecture. Similarly, an imbalance between peripheral tension and core compression can be hypothesized to disrupt the tensegral structure of the microtumor and cause local invasion. These findings therefore suggest that alterations in the tensegral force balance in tissues may play a role in both priming and driving pathogenic phenotypes, particularly in cancer progression.

#### 2. Embedded deformable structures

Integrating deformable structures into an otherwise mechanically complex tissue provides a convenient method to quantify contractile forces, as the mechanical properties of the deformable structures can be well-defined. For example, thin films can be designed to quantify tissue-scale contractile forces of self-assembled myocardial cell sheets by measuring how much they curl up under contractile forces [Figs. 4(d) and 4(e)] and can be readily implemented using a variety of techniques including soft lithography,^167^ spincoating,^148,168^ laser cutting,^169–172^ multi-material 3D-printing,^173^ and microfluidic techniques.^174^ Incorporating grooves on the cantilever's surface aids cell alignment, and generates enhanced contractility.^170,175,176^ This system can be designed to directly readout strains electronically, by incorporating strain gauges within the PDMS (polydimethylsiloxane) films.^171,173^

While this method is limited to measuring tension generation in a sheet of cultured cells, the concept can be extended by designing more advanced structure geometries. Legant *et al.* first developed microfabricated pillars with integrated anchoring structures [Figs. 4(f) and 4(g)],^149^ where pillar deflection can be used to study contractile forces generated across a 3D tissue. Applications to date have included fibroblast-populated collagen matrices,^149^ cardiac tissues,^177–182^ airway smooth muscle and fibroblast co-cultures,^183,184^ wound healing,^185^ clotting under shear flow,^186^ skeletal tissues,^187^ and smooth muscle constructs.^188^ Multi-pillar arrays can also be developed to study directional stresses across the tissue, to study the effects of fibrosis^183^ and breast cancer proliferation [Fig. 4(h)].^189^ In each of these studies, the induced prestress in the self-assembled tissues was found to be crucial for contractile functionality and for generating normal physiological responses. The balance in prestress magnitude also arises as an important parameter, where suboptimal stress can impede maturation, while excessive stresses triggers pathogenic fibrosis and hypertrophy.^190^

Similarly, the deflection of suspended polymer wires during the formation of a tissue can be used to quantify forces across the tissue in a high-throughput well-plate format [Figs. 4(i) and 4(j)]. This system, known as the Biowire II, can be readily integrated with stimulation electrodes to study myocardial fibrotic contraction forces^191^ and can be extended to generate spatially differentiated atrio-ventricular co-cultures to model diseases such as ventricular hypertrophy.^150^ Interestingly, passive tension is higher in fibrotic tissues, impeding their ability to generate sufficient contractility for proper function.^191^ An alternative wire-based platform integrates a flexible plastic probe to apply and monitor local tension to the microtissue by bending the suspended tissue.^192,193^ This platform was used to demonstrate that the tissue prestress was necessary for contraction generation potential^192^ as well as being used to quantify changes in tissue mechanical properties during active and passive force generation.^194^

Recently developed filamentous matrices provide a way to make similar measurements to the Biowire II platform with higher resolution within the tissue since fibers span single cell widths and are compliant under cell-generated forces [Figs. 4(k) and 4(l)]. Matrices composed of organic–inorganic hybrid polymer filaments of varying diameters, synthesized by two-photon photopolymerization methods, are coated in Matrigel, and seeded with cardiomyocytes which self-assemble to form cardiac tissues.^151^ By quantifying the deflection of fibers under cell-generated stresses, the non-uniformity of microenvironmental properties leads to contractile malfunction and disorganization in myocardial tissues.^195^ A fibronectin-derived grid can also be used in a similar fashion draped on top of individual cells, cell sheets, or tissues, where the grid deformations provide reference-free compression and tension quantification.^196^

Internal tissue prestress can also be controlled by designing actuatable structures that apply local forces on demand. By mounting nickel microbeads^197^ or bars for high throughput capacities^198^ onto pillars, cantilevers can be actuated by electromagnetic forces. Alternatively, pneumatically actuated cantilevers can also be designed to provide a wider range of induced strains.^199–201^ Under cyclic loads, the tissue prestress could be manipulated, allowing measurements that decouple the effects of uniaxial^197,202^ and multiaxial^203^ cell-generated forces and ECM properties on tissue stiffening and stabilization.^204^ Notably, microtissues were found to strain-soften and lengthen to maintain their mean tension,^205^ suggesting tissues respond to externally applied stresses by shifting their architecture to maintain tensional homeostasis. While compressive stresses were not quantified in this scenario, we hypothesize that tensegrity exhibits itself as the observed redistribution of forces and consequent tissue reorganization. Taken together, these studies lead to the intriguing hypothesis that the balance between tensegrity-associated forces in prestressed tissue may lead to either homeostasis or pathogenesis when dysregulated.

### B. Micro-scale tissue forces

The techniques discussion in Sec. IV A are limited in their capacity to resolve spatial differences within the microtissues and instead provide readouts of force over the whole tissue. These metrics, hence, provide an ensemble measurement over multiple cells and cannot resolve forces at the single-cell length scale. These limitations make it challenging to observe tensegrity within the microtissues themselves, and the following techniques have recently been developed to provide sufficient spatial resolution to make these inferences.

#### 1. Oil droplets

To achieve high spatial resolution within microtissues, deformable structures must be both small enough and compliant enough to observably deform under cell-generated stresses. Injectable oil microdroplets were first developed and introduced between cells in mammary epithelial aggregates and embryonic tissues to quantify anisotropic forces at the droplet location [Fig. 4(m)].^207,208^ Oil microdroplets could be designed and functionalized to allow cell adhesion, and direct observations of droplet shape allow measurements of local shear stress. Local cellular-generated stresses were found to increase in mesenchymal aggregates due to growth and compaction of the spheroids.^208^ Oil droplets containing magnetic particles can also be magnetically actuated to locally probe mechanical tissue. These studies reveal cell-scale viscoelastic properties in zebrafish embryos, which play a key role in time-dependent elongation of the tissue.^209^ Furthermore, cell jamming was found to yield more solid-like tissue, providing a backbone during embryonic elongation, while other regions of the tissue remained fluid-like and remodeled under supracellular-generated stresses.^210^ These mechanical insights into the morphogenesis process suggest that an intricately organized tensegral system of sequentially stabilized units arises to develop form and function from a relatively homogenous embryo.

#### 2. Deformable hydrogel particles

Oil microdroplets are incompressible and can only be used to quantify anisotropic forces. In contrast, deformable hydrogel beads can be both porous and compressible, thus offering a way of quantifying absolute stresses within tissues [Figs. 4(n) and 4(o)]. The stiffness of polyacrylamide,^152,211–214^ poly(ethylene glycol),^215^ and alginate^216^ beads can be tuned to measure a range of forces, from applied compressive stresses^211^ to cell-generated tensile stresses within fibroblast spheroids.^152^ While certain sensors rely on pressure-induced fluorophore diffusion^213^ or local FRET-based changes in fluorescence due to deformations^215^ to quantify stresses, they require extensive characterization and calibration to be used *in vivo*. Tracking the deformation of beads within multi-layered cell sheets,^216^ spheroids,^152,211,216^ and zebrafish embryos^214,216^ has been shown to resolve tissue pressure and cell-generated force profiles throughout tissues and development. Lee *et al.* used this system to demonstrate that a “skin” of tension forms on the periphery, while compressive stresses build up towards the core of fibroblast spheroids [Fig. 4(o)]. Inhibition of peripheral contractility also decreased spheroid compaction, indicating that outer tension is counterbalanced by compression of cells at the core, and demonstrating that observable tensegral patterns arise within days of spheroid formation.^152^ More recently, thermoresponsive hydrogel droplets made of poly-n-isopropylacrylamide swell on demand and can be used to probe local stiffness within tissues by comparing the swollen and compacted states of the hydrogel droplets. This technology was used to demonstrate that significant spatial heterogeneities exist in invasive breast cancer tissue mechanics.^217^ These findings demonstrate that, even though tensegrity might exist to stabilize the tissue, local mechanical properties arise to internally stabilize and prime the spheroid for future biological activity.

## V. EMERGING VIEWS OF TENSEGRITY IN BIOLOGY

Spatial force profiles within tissues, cells, and molecules are essential in dictating the progression of tissue shape and function in homeostasis, morphogenesis, and pathogenesis. During development, cell-generated forces are well-established to drive key changes in tissue configurations, such as gastrulation,^6^ epithelial curling,^218^ and elongation.^9^ The compressional and tensile balances of forces within the tissue, as well as their spatial configuration, are essential for the proper tissue formation in such processes.^7^ The balance of these forces in hierarchical, networked architectures also allows complex deformation modes such as shear at larger length scales, despite not including shear elements in the tensegral formulation. Local force coordination through cytoskeletal components like microtubules^10^ and long-range force transmission through fiber strain-stiffening^219^ further emphasize the importance of tensegral prestress in tissue formation, without which, cohesive behavior of cells within a tissue would be severely impaired. Not only is this coordination of forces and tissue prestress key during morphogenesis but also during wound healing to orchestrate collective cell migration^13^ and initiate wound closure.^206^

While classical tensegrity structures are composed of both rigid (load-bearing) and linearly elastic components that return to their initial shape after external loads are removed, biological tissues do not exhibit this general behavior. Instead, the elastic response is typically observed immediately after mechanical load, and viscoelastic behavior dominates over the long-term, leading to creep, release of internal stress, and plastic or permanent remodeling. When stretched, both cells^129^ and tissues^205^ strain-soften over time, resulting in decreased prestress to preserve their tensional balance. During morphogenesis, this process of viscoelastic remodeling is essential, as the tissue must undergo strain-softening and plastic deformation to adopt the shapes necessary for tissue and organ function.^209^ To guide this shape change, active and rigid mechanical elements must also play a role. For example, jammed cells can form solid-like regions within the tissue, providing mechanical support to bear the compressive load required to elongate the tissue.^210^ Thus, both static and dynamic force balances in viscoelastic tissues ensure proper formation and remodeling, especially in morphogenesis. Hence, incorporating time-dependent elements in tensegrity models may be an important strategy to model longer-term developmental processes.

The role of tensegrity in pathological processes is less well-established and may prove to be a novel line of inquiry in understanding the mechanics of disease progression. In luminal cancers, such as breast carcinomas, the tissue begins as a hollow duct structure. The basal side on the outside of the duct and the apical side on the inside of the duct can both be considered as tensile skins balanced by the pressurized fluid within the lumen. Luminal collapse on the apical side is a structural failure which occurs in early breast cancer progression,^220^ suggesting that a significant imbalance between lumen compression as well as basal and apical tension leads to the cancer's progression. We reason that once the luminal region begins to fill with cells at the onset of ductal hyperplasia, the duct solidifies and develops additional growth-induced compressive stresses, and tension in the basal layer and myoepithelium must be released and breached during structural progression of the disease. Interestingly, cell extrusion by overcrowding in epithelial tissues has also been suggested as a mechanism for luminal filling in cancer, which normally occurs apically to maintain epithelial homeostasis, but occurs basally in more aggressive diseased conditions.^221–223^

The importance of tensegrity during pathological processes may be more directly considered by correlating the invasive properties of cells with their spatial distribution within tumors. Cells at the periphery of breast cancer spheroids are softer and more motile than those under compressional load at the core. This compressive load is hypothesized to push intracellular fluid through intercellular gap junctions, effectively swelling cells under less compressive stresses, yielding a more invasive phenotype at the periphery.^224^ Following the tensegrity model, higher compressive stresses at the core would require higher tensile stresses at the periphery to stabilize the structure. Interestingly, generating these higher tensile stresses would require the peripheral cells to be more contractile, and this cell phenotype is known to remodel and reorganize collagen, stiffening local ECM and facilitate local invasion of cancer cells.^15^ This suggests that cells at the periphery might be more contractile due to the build-up of compression at the core, effectively highlighting the importance of tensile and compressive force balances in the tissue. Additionally, the cells at the core were less active and motile,^224^ which is consistent with the concept of increased cell jamming under compressive stresses from neighboring cells, which must overcome higher energy barriers to migrate past each other.^225^ The unjamming process seems to play a key role in breast cancer progression, where the decrease in compressive stresses enhances collective migration in more invasive cancers.^166^

**FIG. 5.**
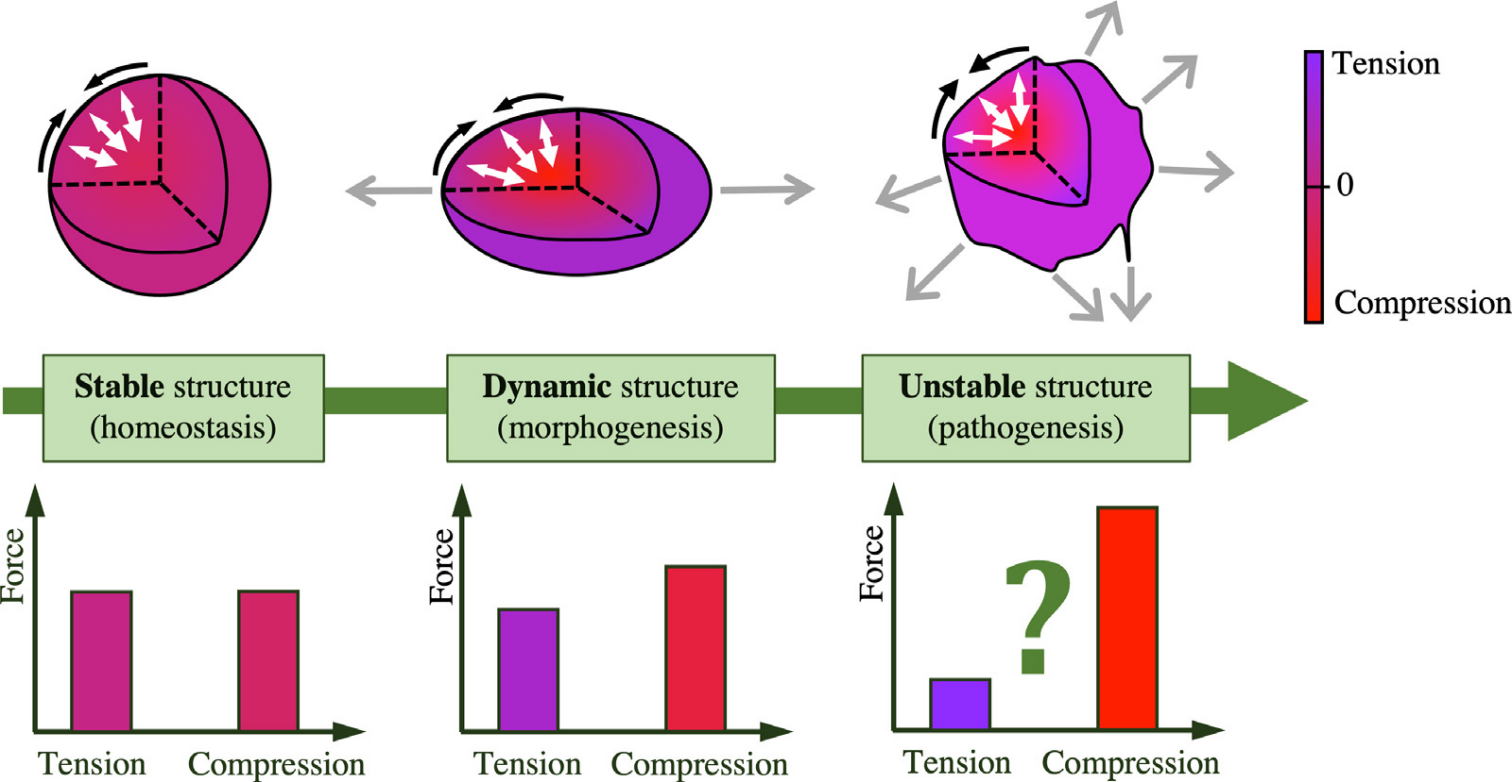
The tensegrity spectrum: stable, dynamic, and unstable structures. When compressive and tensile stresses are balanced with the appropriate magnitude, the overall tensegral tissue structure is stable, a necessary condition for homeostasis. Dynamic structures arise with slight imbalances in stresses, as seen in morphogenesis; while we theorize that an extreme imbalance in stresses can lead to pathogenesis and disease progression. Black arrows indicate peripheral tensile stresses, white arrows indicate growing compressive stresses towards the core, and gray arrows indicate shape changes.

Taken together, this mechanical analysis of disease progression suggests that when the balance between tensional and compressional stresses is affected, this can lead to disease progression. We propose that the normal, developmental, and pathological balance between tensegral force balances range across a spectrum (Fig. 5). A perfect dynamic balance between tension and compression provides a stable biological structure that maintains homeostatic function. Small imbalances, in conjunction with time-dependent changes in tissue mechanical properties, provides the mechanical conditions to drive shape and function changes during morphogenesis. We theorize that extensive imbalances compromise the tissue's tensegrity, leading to structural instability and loss of organization, resulting in disease progression such as unrestricted growth and local invasion in cancer, or loss of architecture and function in degenerative disease conditions. Hence, developing a tensegrity index to quantify these imbalances across multiple length scales and spatial locations may be of considerable value in robustly predicting and interfering with disease progression. This would be particularly challenging as a tensegrity index would have to simultaneously convey quantitative information comparing tensional and compressional signatures as a function of space, and of hierarchical length scale. Formulating such an index might, however, facilitate distinguishing between homeostatic, dynamic, and unstable biological structures (Fig. 5). Considerable advances in both biomaterials-based techniques to measure forces and local mechanics within realistic tissue models, as well as a common language and descriptor for the mechanical state of tissues that captures time-, space-, and state-dependent properties and progression of the tissue will, therefore, be needed to apply this concept for practical gain.

## VI. CONCLUSIONS

Cell-generated forces have been quantified across the biological length scales from the molecular to the tissue level by engineering designer biomaterials to provide insight into the location, direction, and magnitude of these forces. Molecular probes have uncovered patterns in tensile stresses within single cells and cell–cell junctions, highlighting the importance of cytoskeletal prestress for cell shapes and functions. Quantifying forces at the cellular level further confirms the significance of internal prestress within cells in order to exert forces on their surroundings and also develops a better understanding of mechanotransductive pathways involved in cell signaling and disease. Novel developments to measure these forces in highly realistic 3D engineered tissues further suggest that tensegrity is a fundamental, modular, and hierarchical characteristic across biological length scales. These studies confirm and reinforce the ubiquitous presence of tensegrity as an organizing principle for biological systems. Given the accumulating evidence that tissue stability, morphogenesis, and pathogenesis of several diseases and organ systems are associated with tensegral imbalances, there remains a fundamental need to further develop the techniques necessary to measure these complex features of tissues in development and disease.

## Data Availability

Data sharing is not applicable to this article as no new data were created or analyzed in this study.

## NOMENCLATURE

ATP: Adenosine triphosphate
DNA: Deoxyribonucleic acid
ECM: Extracellular matrix
FRET: Fluorescence resonance energy transfer
RGD: Arginine–glycine–aspartate
TFM: Traction force microscopy
